# 3D-Printed vs Commercial Distal Caps for ESD: Multicenter Randomized *Ex Vivo* Noninferiority Trial (ENDOPRINT Trial)

**DOI:** 10.14309/ctg.0000000000001022

**Published:** 2026-03-23

**Authors:** Jan Kral, Daryl Ramai, Jakub Svet, Vit Navratil, Katerina Knotkova, Aneta Sikynova, Abdulrahman Qatomah, Jakub Sobotka, Hiroyuki Aihara, Lumir Kunovsky

**Affiliations:** 1 Department of Internal Medicine, Motol University Hospital and Homolka, Second Faculty of Medicine, Charles University, Prague, Czech Republic;; 2 3NDO, 3D-Printing Technological Start Up, Prague, Czech Republic;; 3 Division of Gastroenterology, Hepatology, and Endoscopy, Brigham and Women's Hospital, Harvard Medical School, Boston, Massachusetts, USA;; 4 Division of Gastroenterology, Hepatology, and Nutrition, University of Utah, Salt Lake City, Utah, USA;; 5 2nd Department of Internal Medicine—Gastroenterology and Geriatrics, University Hospital Olomouc, Faculty of Medicine and Dentistry, Palacky University Olomouc, Olomouc, Czech Republic;; 6 Institute of Biostatistics and Analyses, Faculty of Medicine, Masaryk University, Brno, Czech Republic;; 7 Department of Surgery, University Hospital Brno, Faculty of Medicine, Masaryk University, Brno, Czech Republic; 8 Department of Gastroenterology and Digestive Endoscopy, Masaryk Memorial Cancer Institute, Brno, Czech Republic.

**Keywords:** ESD, CAP, endoscopy

## Abstract

**INTRODUCTION:**

Distal attachment caps enhance visualization and traction during endoscopic submucosal dissection (ESD) but may influence procedural ergonomics. We conducted the multicenter ENDOPRINT randomized comparative feasibility study with a prespecified noninferiority hypothesis to compare a custom 3D-printed cap made from flexible medical-grade resin with a standard commercial cap using an *ex vivo* porcine model across tertiary centers in Prague, Olomouc, and Boston.

**METHODS:**

Across 3 centers, 99 *ex vivo* ESD procedures (33 per site) were randomized 1:1 to a 3D-printed cap or a commercial Olympus cap. The primary end point was procedure time; secondary end points included lesion size, en bloc resection, technical success, ergonomic parameters (visibility, scope manipulation, tissue retraction), cap handling, and adverse events. Group allocation was balanced across sites.

**RESULTS:**

Median procedure time was identical for both caps (17.3 minutes each; *P* = 0.952), remaining well within the predefined 20% noninferiority margin, and lesion sizes were comparable between groups. En bloc resection and technical success were achieved in 100% of cases in both arms. Ergonomic assessments favored the 3D-printed cap, with higher operator ratings for visibility, manipulation, and tissue retraction, all with *P* < 0.001 and consistent across all centers. Adverse events were uncommon and similar between groups. Procedure time correlated with lesion size (*P* = 0.001).

**DISCUSSION:**

The ENDOPRINT trial demonstrated that a 3D-printed distal cap is as efficient and safe as a commercial cap during ESD, while providing superior visibility, scope control, and tissue traction. These results support the potential of customizable 3D-printed accessories with substantially lower per-unit material costs compared with commercial devices for clinical practice and training, warranting further *in vivo* validation and formal cost-effectiveness studies.

## Graphical abstract

**Figure:**
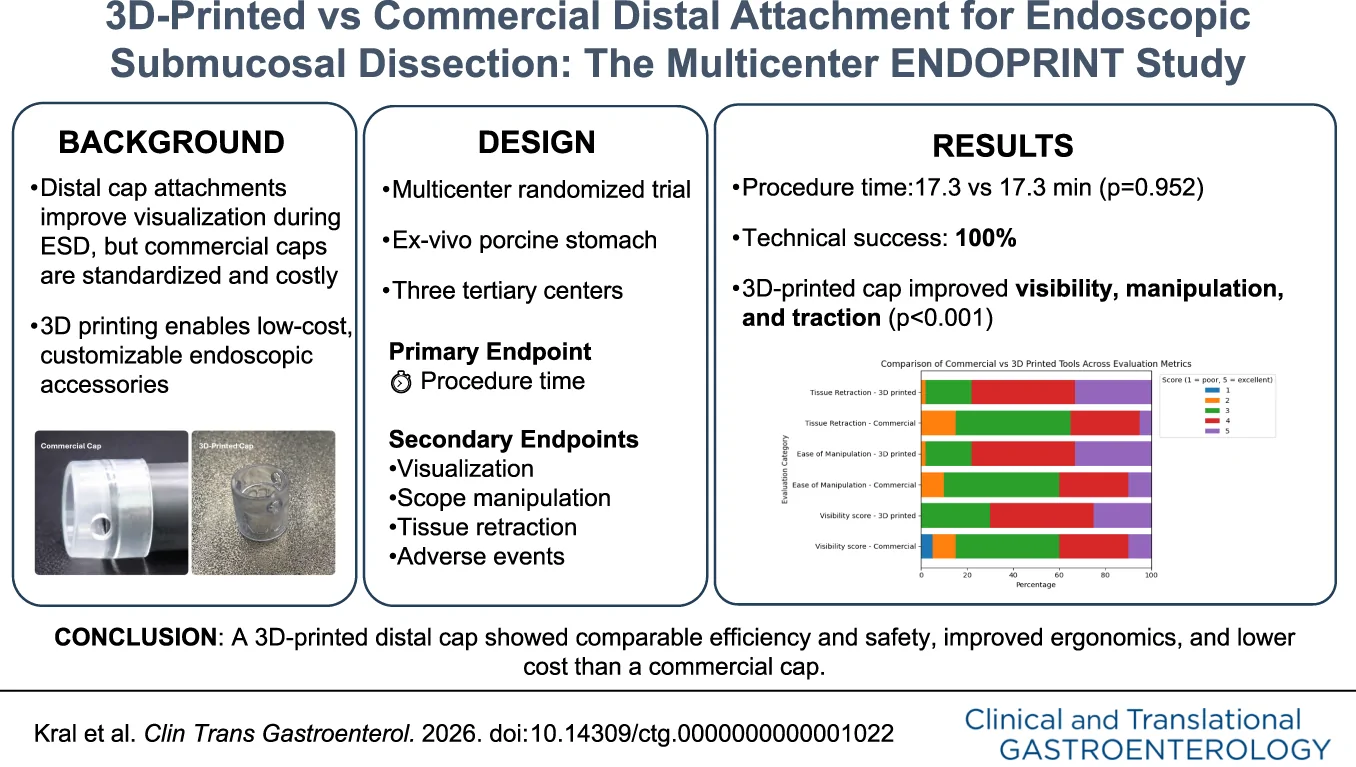

## INTRODUCTION

Endoscopic submucosal dissection (ESD) has emerged as a cornerstone technique for achieving *en bloc* resection of superficial neoplastic lesions in the gastrointestinal tract (1). Its adoption has grown significantly, particularly in East Asia, and is gaining traction in Western centers owing to its superior histopathological and oncological outcomes compared with conventional endoscopic mucosal resection (2,3). However, ESD remains a technically demanding and time-intensive procedure, often associated with steep learning curves and a risk of adverse events such as perforation and bleeding (4,5).

One of the critical facilitators of effective ESD is the use of a distal attachment (cap) mounted on the endoscope tip. This accessory improves visualization of the submucosal plane, enables dynamic tissue traction, and aids in maintaining a stable field during dissection. Commercially available caps, such as those provided by Olympus or FujiFilm and other manufacturers, are typically standardized in size and curvature. Although generally effective, their rigid, one-size-fits-all design may not accommodate variations in lesion morphology, endoscope models, or procedural nuances. In addition, the cost and limited adaptability of commercial caps pose challenges for centers in resource-constrained settings or those engaged in iterative procedural innovation (6).

Recent advances in 3-dimensional (3D) printing technology have opened novel avenues for custom device prototyping in interventional endoscopy (7,8). 3D printing enables rapid, low-cost fabrication of anatomical and procedural tools with customizable design features. Its application in endoscopic training models, stents, and accessories has demonstrated feasibility and promise (9,10). However, to date, there have been no direct comparative studies evaluating the use of 3D-printed caps in ESD procedures against commercially available caps.

In this study, we designed and fabricated a novel 3D-printed cap tailored to fit a standard therapeutic gastroscope, with specific attention to optimizing visual field, tissue manipulation, and procedural ergonomics. We hypothesized that the 3D-printed cap would be noninferior to the commercial Olympus cap regarding procedural performance, particularly resection time, in an *ex vivo* porcine model. This prospective, randomized, comparative feasibility study was designed to test a noninferiority hypothesis comparing resection time, visualization, ease of manipulation, tissue retraction, and adverse event proxies between the 2 cap types. This study represents the first preclinical investigation directly comparing 3D-printed and commercial cap in a controlled ESD setting, providing preliminary evidence to support the use of customizable printed devices as cost-effective, adaptable alternatives for therapeutic endoscopy.

## METHODS

This was a prospective, randomized, head-to-head feasibility study designed to compare the procedural performance of a novel 3D-printed cap with a standard commercial Olympus cap (commercial) during ESD in a porcine model. The ENDOPRINT trial was conducted at 3 sites (Prague and Olomouc, Czech Republic; Boston, MA) using *ex vivo* pig stomachs. Each stomach had a 20 mm artificial lesion marked and underwent ESD using a randomized cap (Commercial or 3D-printed). Artificial lesions were created in technically accessible gastric regions, predominantly in the antrum and along the anterior wall, to ensure procedural standardization and reproducibility across centers. Lesion location was not formally randomized but selected to maintain comparable technical conditions between procedures. Centers used identical procedural protocols, including Olympus DualKnife and submucosal injection with saline/methylene blue. The custom 3D-printed caps were developed using computer-aided detection software (Autodesk Fusion 360, Autodesk Inc) to precisely match the distal dimensions of a gastroscope (Olympus GIF- HQ190 or EZ1500). The design featured a 15° oblique-cut leading edge to enhance submucosal visualization and tissue manipulation. The obliquely cut distal surface was oriented such that the shorter edge was aligned toward the endoscopic lens, whereas the longer edge was positioned opposite the lens axis, optimizing forward visualization and minimizing lens shadowing during submucosal dissection. In addition, the 3D-printed cap incorporated 3 evenly distributed lateral side holes circumferentially positioned along the cap body. These openings were designed to facilitate fluid and debris drainage, reduce suction-related mucosal adherence, and maintain a clear visual field during ESD (Figure 1). This design differs from the conventional commercial cap, which features a symmetrical, nonangulated distal rim and lacks multiple lateral side holes. For the purposes of this study, all 3D-printed caps were produced using an identical standardized design and were not personalized for individual lesions. The potential for device customization represents a conceptual advantage of 3D printing technology but was not specifically evaluated in this experimental model. Caps were fabricated using a high-resolution stereolithography 3D printer (Formlabs Form 3B, Formlabs Inc) with a medical-grade biocompatible photopolymer resin (BioMed Flex 80A Resin, Formlabs Inc), using a layer resolution of 50 μm and full infill to maximize rigidity. The commercial cap served as the control and was used in a standardized manner. Operators could not be blinded because of cap visibility; however, data assessment was standardized across centers. A total of 99 procedures were performed. All procedures were performed by experienced endoscopists trained in ESD. Although all operators were proficient in ESD technique, procedural volume differed between centers. The Boston center represented a high-volume ESD practice, whereas Prague and Olomouc reflected lower-volume Western programs. Within each center, cap allocation was randomized 1:1, ensuring balanced device comparison at the operator level.

**Figure 1.**
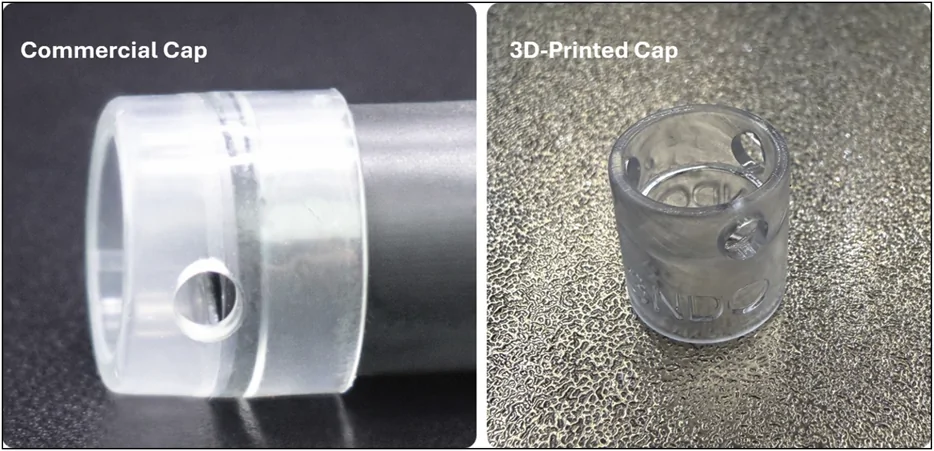
Commercial cap vs 3D-printed cap.

### Outcome measures

The primary outcome was procedure time, defined as the interval from the initial mucosal incision to the completion of en bloc resection, measured in minutes. This served as the principal indicator of procedural efficiency. Secondary outcomes included lesion size (in millimeters), visualization quality, ease of manipulation, and tissue retraction, each rated by the operator on a 5-point Likert scale (11). Procedural handling was further evaluated by documenting the number of cap exchanges or adjustments and the frequency of cap cleaning because of visual obstruction. Technical success was defined as the completion of the ESD procedure using the assigned cap without switching devices. En bloc resection was confirmed macroscopically, and adverse event proxies, including visible serosal tears or perforations, were recorded immediately after each procedure. All data were collected prospectively using standardized case report forms.

### Statistics

Statistical analyses were performed using standard descriptive methods. For continuous data, mean, median, SD, minimum, and maximum were reported. Comparisons of continuous outcomes between cap groups (3D-printed vs commercial) used the Wilcoxon rank-sum test. Categorical and ordinal variables were summarized as frequencies and percentages, and group differences were assessed using the Fisher exact test. Associations between continuous variables, such as lesion size and procedure time, were measured using the Spearman correlation coefficient.

A *P*-value less than 0.05 was considered statistically significant for all comparisons, with analyses based on per-protocol data. This approach was consistent across all key end points, ensuring robust and reproducible evaluation of device performance and procedure characteristics.

A noninferiority hypothesis was defined with procedure time as the primary end point. A difference exceeding 20% of the median procedure time observed with the commercial cap was considered clinically relevant. Based on a reference median time of approximately 17 minutes, this corresponded to a noninferiority margin of 3.4 minutes.

As this was a multicenter preclinical feasibility study, a formal a priori sample size calculation was not performed. The sample size of 99 procedures (33 per center) was determined pragmatically to ensure balanced representation across sites and to provide sufficient precision for estimation of procedural performance and variability.

## RESULTS

A total of 99 lesions simulated using *ex vivo pig stomachs* were randomized and analyzed from 3 centers (Prague, Olomouc, Boston), with 33 procedures performed per site. Lesion location was comparable across centers, as lesions were predominantly created in accessible gastric regions (antrum/anterior wall), minimizing variability in technical difficulty. Of these, 51 ESDs used a 3D-printed cap (52%) and 48 used a commercial cap (48%). The distribution of cap types was statistically similar across all centers (3D-printed vs Commercial: Olomouc 20/13, Prague 15/18, Boston 16/17; *P* = 0.446), confirming equivalent group allocation and minimizing selection bias.

Median lesion size for the entire cohort was 22.6 mm (range 15–40 mm). When compared between groups, the 3D-printed cap had a median lesion size of 23.5 mm, and Commercial 21.7 mm, with no significant difference (*P* = 0.165). Median procedure time was 17.3 minutes overall. Procedure times were identical between cap types (3D-printed: 17.3 minutes, Commercial: 17.3 minutes; *P* = 0.952; Table 1, Figure 2). Notable center-level differences were observed. Boston exhibited a significantly shorter median procedure time (∼5 minutes), whereas Prague and Olomouc results clustered around the cohort median (∼20 minutes). This difference likely reflects variation in operator experience and institutional workflow efficiency across centers. Importantly, lesion size distribution remained consistent between sites, and cap allocation was balanced within each center through 1:1 randomization. Multivariate analysis confirmed that procedure time was significantly influenced by center (*P* = 0.001), whereas cap type was not an independent predictor of procedural duration. These findings suggest that intercenter variability was driven by operator and workflow factors rather than device-related performance differences.

**Table 1.** Lesion and procedure times characteristics

Characteristics	N (%)	Mean (SD)	Median (min–max)	*P*-value (Wilcoxon rank-sum test)
Lesion size (mm)	99 (100%)	22.6 (5.5)	20 (15–40)	0.165
Commercial	48 (48%)	21.7 (5.0)	20 (15–35)	
3D printed	51 (52%)	23.5 (5.9)	21 (15–40)	
Procedure time (min)	99 (100%)	17.3 (10.9)	21 (2–45)	0.952
Commercial	48 (48%)	17.3 (11.4)	21 (2–45)	
3D printed	51 (52%)	17.3 (10.6)	20 (2–45)	

**Figure 2.**
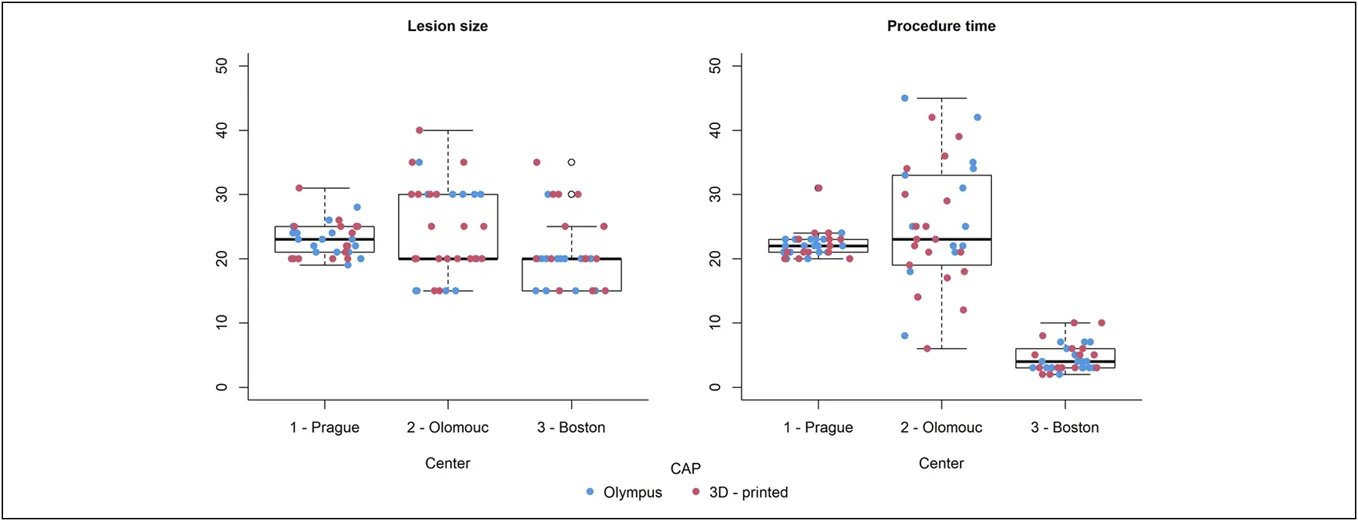
Lesion size and procedures times by center.

Subjective evaluations by operators were quantified using 5-point Likert scales for visibility, ease of manipulation, and tissue retraction. Use of the 3D-printed cap yielded significantly higher scores in all 3 domains: visibility (median score 4 vs 3; *P* = 0.001), ease of manipulation (4 vs 3; *P* = 0.001), and tissue retraction (5 vs 3; *P* = 0.001) (Figure 3). Notably, excellent tissue retraction (score of 5) was achieved in 33 ESDs using the 3D-printed cap compared with only 4 with Commercial cap. Cleaning requirements, cap exchanges/adjustments, technical success (defined as completion without device switching), and *en bloc* resection rates were identical and optimal in both arms (100% success and *en bloc* for all cases). Adverse events, including minor tears and perforation, were rare and did not differ between groups (tears: 4 vs 3 cases; perforation: 0 vs 1 case for Commercial cap and 3D-printed cap, respectively) (Table 2).

**Figure 3.**
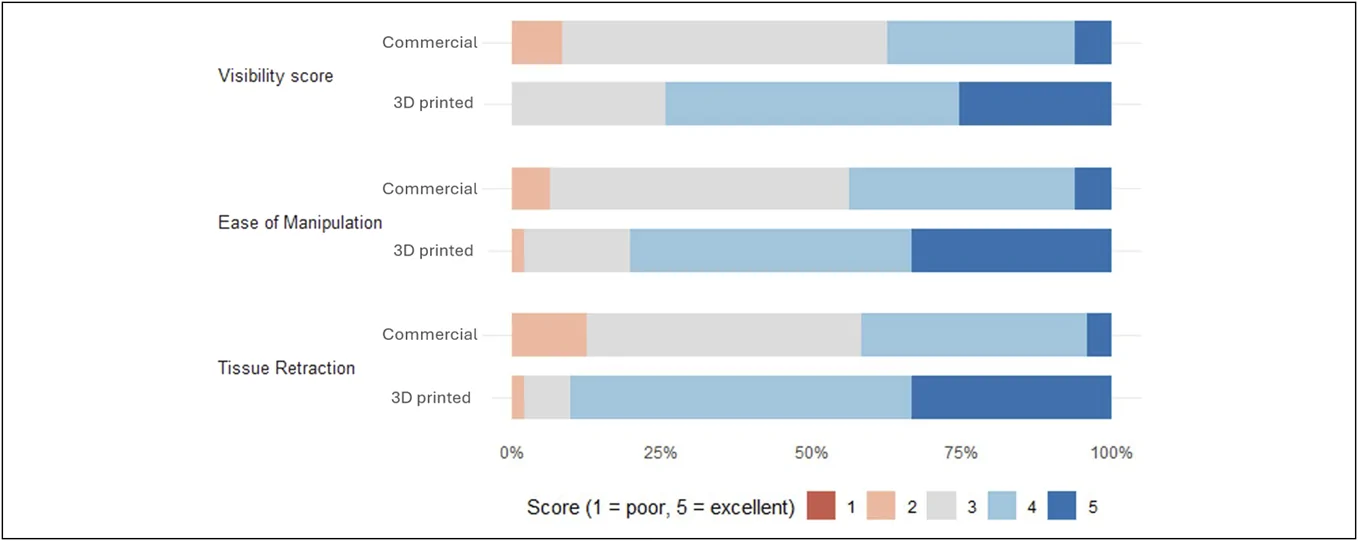
Differences in metrics.

**Table 2.** Procedural and performance characteristics by cap type

Characteristics	Total N (%)	Commercial N (%)	3D printed N (%)	*P*-value (the Fisher exact test)
Visibility score	99 (100%)			<0.001
1 (poor visualization)	0 (0%)	0 (0%)	0 (0%)	
2	4 (4%)	4 (8%)	0 (0%)	
3	39 (39%)	26 (54%)	13 (25%)	
4	40 (40%)	15 (31%)	25 (49%)	
5 (excellent visualization)	16 (16%)	3 (6%)	13 (25%)	
Ease of manipulation	99 (100%)	48 (48%)	51 (52%)	<0.001
1 (very difficult)	0 (0%)	0 (0%)	0 (0%)	
2	4 (4%)	3 (6%)	1 (2%)	
3	33 (33%)	24 (50%)	9 (18%)	
4	42 (42%)	18 (38%)	24 (47%)	
5 (very easy)	20 (20%)	3 (6%)	17 (33%)	
Tissue retraction	99 (100%)	48 (48%)	51 (52%)	<0.001
1 (minimum retraction)	0 (0%)	0 (0%)	0 (0%)	
2	7 (7%)	6 (13%)	1 (2%)	
3	26 (26%)	22 (46%)	4 (8%)	
4	47 (47%)	18 (38%)	29 (57%)	
5 (excellent retraction)	19 (19%)	2 (4%)	17 (33%)	
Distal attachment exchanges/adjustments	99 (100%)	48 (48%)	51 (52%)	
0	99 (100%)	48 (100%)	51 (100%)	
Distal attachment cleaning	99 (100%)	48 (48%)	51 (52%)	0.141
0	86 (87%)	39 (81%)	47 (92%)	
1	13 (13%)	9 (19%)	4 (8%)	
Technical success	99 (100%)	48 (48%)	51 (52%)	
Yes	99 (100%)	48 (100%)	51 (100%)	
En block resection	99 (100%)	48 (48%)	51 (52%)	
Yes	99 (100%)	48 (100%)	51 (100%)	
Adverse events	99 (100%)	48 (48%)	51 (52%)	0.852
None	91 (92%)	44 (92%)	47 (92%)	
Tears in muscular layer	7 (7%)	4 (8%)	3 (6%)	
Perforation	1 (1%)	0 (0%)	1 (2%)	

Lesion size showed a moderate positive correlation with procedure time (ρ = 0.44; *P* = 0.001), indicating that larger lesions typically required longer procedures across both cap types. Statistical analysis confirmed that procedure time was significantly influenced by the center (*P* = 0.001), rather than by device type. Ergonomic factors including ease of manipulation, visibility, and tissue retraction were strongly associated (all *P* = 0.001), reflecting the interrelated nature of operator perception of procedural ergonomics during ESD. Table 3 presents correlation matrices calculated for the entire cohort (Total) and separately within each cap group (“Commercial” and “3D printed”). The analysis demonstrated consistent relationships between ergonomic parameters across both cap types, with improvements particularly notable in the 3D-printed cap group. Safety analysis demonstrated no significant associations between any assessed factor and adverse event rates, supporting an equivalent safety profile for both caps (Table 3). Multivariate analysis confirmed that cap type did not independently predict procedure time, adverse events, or procedural success after accounting for lesion size and center (all *P* > 0.05). Stratified analysis revealed that 3D-printed caps offered procedural advantages (visibility, manipulation, tissue retraction) regardless of center, but overall procedural speed remained most affected by site-level workflow (Table 3).

**Table 3.** Correlation matrix of procedural and ergonomic parameters

	Center	Visibility score	Ease of manipulation	Tissue retraction	Distal attachment cleaning	Adverse events
Center						
Total		0.063	0.002	0.174	0.012	1
Commercial		0.159	0.047	0.193	0.044	1
3D printed		0.054	<0.001	0.057	0.061	0.663
Visibility score						
Total	0.063		<0.001	0.001	0.589	0.601
Commercial	0.159		0.078	0.167	0.701	0.624
3D printed	0.054		0.031	0.495	0.797	0.719
Ease of manipulation						
Total	0.002	<0.001		<0.001	0.740	0.964
Commercial	0.047	0.078		0.007	0.407	1
3D printed	<0.001	0.031		0.041	0.543	0.812
Tissue retraction						
Total	0.174	0.001	<0.001		0.444	0.198
Commercial	0.193	0.167	0.007		0.164	0.261
3D printed	0.057	0.495	0.041		1	0.498
Distal attachment cleaning						
Total	0.012	0.589	0.740	0.444		0.643
Commercial	0.044	0.701	0.407	0.164		1
3D printed	0.061	0.797	0.543	1		1
Adverse events						
Total	1	0.601	0.964	0.198	0.643	
Commercial	1	0.624	1	0.261	1	
3D printed	0.663	0.719	0.812	0.498	1	

Total represents correlations calculated across the entire cohort, whereas commercial and 3D printed represent correlations calculated within each cap group separately.

A preliminary cost assessment was performed. The BioMed Flex 80A resin costs approximately 249 USD per liter. Given that approximately 1 mL of resin is required per cap, the estimated material cost per 3D-printed cap is ∼0.25 USD. Printing time per production batch is 2 hours and 33 minutes and is independent of the number of caps produced in a single run. In comparison, a commercial distal attachment cap costs approximately 38 USD per unit. Although stereolithography printing requires an upfront equipment investment (Formlabs Form 3B ∼6,000 USD), the per-unit material cost of the printed device is substantially lower. A formal cost-effectiveness analysis was beyond the scope of this preclinical study.

## DISCUSSION

This multicenter randomized study demonstrates that 3D-printed distal attachments perform comparably to available commercial caps in ESD within an *ex vivo* porcine model. Our findings support the noninferiority hypothesis because the mean resection time using the 3D-printed cap was statistically equivalent to that of the commercial cap, with no significant difference in adverse event proxies.

The primary outcome did not differ significantly between the groups (17.3 minutes, *P* = 0.952). These findings mirror those from previous reports exploring cap-assisted ESD optimization, where performance gains are largely driven by improved visualization and maneuverability rather than brand or material of the cap. Oyama et al introduced ESD with a hook knife to enable safe *en bloc* resection of large superficial esophageal lesions. Their findings demonstrated that advanced ESD techniques, with optimized device design and refined visualization, make large *en bloc* resections possible without increasing surgical risks (2).

Endoscopist ratings in our study indicate that the 3D-printed cap provided significantly higher satisfaction for visualization, ease of manipulation, and tissue retraction compared with the commercial cap (secondary outcomes, < 0.001).In addition, technical handling, including cap exchange and cleaning frequency, was equivalent between devices, confirming that the improved subjective experience did not come at the cost of practical usability. These findings are in line with the study by Ko et al, who demonstrated that custom 3D-printed endoscopic caps enabled wider fields of view and better lesion access when compared with standard designs (12). Correlation analysis further demonstrated strong relationships between ergonomic parameters such as visibility, manipulation, and tissue retraction, suggesting that these operator perceptions represent interconnected aspects of procedural ergonomics rather than independent variables. Recent comprehensive review by Zhang et al (7) further emphasize the disruptive potential of 3D printing in gastrointestinal endoscopy, highlighting benefits in adaptability, cost, and performance.

The complication analysis based on observed serosal tears or perforation in our study showed no meaningful difference between groups. Like Saito et al (4) in their review, which found that the rates of serious complications such as perforation and bleeding in gastric ESD are low when the procedure is performed by experienced endoscopists following standardized protocols. Our results confirm that introducing 3D-printed distal caps does not compromise safety, at least in controlled preclinical settings.

Our results have important implications for low-resource settings and innovation hubs. Although the upfront investment in 3D printing infrastructure is non-negligible, the ability to customize devices to specific anatomical or procedural needs may democratize access to high-quality endoscopy and foster rapid iteration during device development. Furthermore, in the context of ESD training, lower-cost, disposable, yet functionally equivalent caps may enhance simulation fidelity without the financial burden of using commercial tools (13). A preliminary cost comparison demonstrated markedly lower per-unit material costs for 3D-printed caps compared with commercially available devices (∼0.25 USD vs 38 USD per unit), although comprehensive health-economic modeling remains necessary.

The observed intercenter variability in procedure time likely reflects differences in operator experience and institutional workflow rather than device performance. However, the randomized allocation within each center preserved internal validity for cap comparison.

This was an *ex vivo* trial using pig stomachs, which lacks the blood flow, motility, and tissue properties of clinical human procedures, so findings may not fully reflect performance in actual patients. Operators were not blinded to cap type because of visible design differences, introducing the possibility of expectation bias in subjective ratings such as visibility and manipulation. Although all participating endoscopists were experienced in ESD, differences in procedural volume between centers could influence ergonomic perception. Importantly, stratified analyses demonstrated that the superior ergonomic ratings associated with the 3D-printed cap were consistent across all centers, including both high-volume and lower-volume practices. Objective procedural end points remained identical between groups. Finally, porcine tissue differs from human tissue, so device performance and safety cannot be directly extrapolated. These factors support the need for future *in vivo* and clinical studies to validate the results and assess cost-effectiveness in real-world practice. In addition, lesion location was not randomized and was preferentially created in technically accessible gastric regions, which may limit generalizability to more challenging anatomical sites.

In conclusion, custom 3D-printed caps proved equally efficient and safe as commercial caps for ESD, while providing better visibility, handling, and tissue traction. These ergonomic benefits were consistent across all centers, supporting 3D-printed caps as practical, customizable, and cost-effective tools for advanced endoscopy. Further *in vivo* studies are warranted to confirm clinical effectiveness.

## CONFLICTS OF INTEREST

**Guarantor of the article:** Jan Kral, MD, PhD.

**Specific author contributions:** J.K.: conceptualization and design of the study; development of the 3D-printed distal attachment; coordination of the multicenter project; performance of ESD procedures; data acquisition and interpretation; drafting of the manuscript; approved the final version of the manuscript. D.R.: contribution to study design and methodology; supervision of the Boston study site; performance of ESD procedures; data acquisition and interpretation; critical revision of the manuscript for important intellectual content; approved the final version of the manuscript. J.S.: technical development and optimization of the 3D-printed cap design; engineering support for device prototyping; performance of ESD procedures; contribution to data collection and interpretation; manuscript review; approved the final version of the manuscript. V.N.: participation in data acquisition at the Olomouc study site; performance of ESD procedures; contribution to data interpretation; manuscript review and editing; approved the final version of the manuscript. K.K.: data collection and organization; coordination of study documentation; contribution to data interpretation; manuscript editing; approved the final version of the manuscript. A.S.: statistical analysis; data validation; interpretation of statistical results; preparation of tables and figures; critical review of the manuscript; approved the final version of the manuscript. A.Q.: participation in procedural execution and data acquisition at the Boston study site; performance of ESD procedures; contribution to data interpretation; manuscript review; approved the final version of the manuscript. J.S.: engineering support for device development; technical validation of the 3D-printed cap; contribution to study methodology; manuscript review; approved the final version of the manuscript. H.A.: senior supervision of study conduct; methodological guidance; interpretation of results; critical revision of the manuscript for scientific content; approved the final version of the manuscript. L.K.: supervision of the Olomouc study site; contribution to study conception and design; performance of ESD procedures; interpretation of results; critical revision of the manuscript; approved the final version of the manuscript.

**Financial support:** None to report.

**Potential competing interests:** J.K.: designer at 3NDO. J.S.: engineer at 3NDO.

**AI:** During the preparation of this work, the author(s) used ChatGPT (OpenAI) solely to check spelling and improve grammatical correctness.

**Study Highlights**

**WHAT IS KNOWN**

✓ Distal attachment caps facilitate visualization and traction during endoscopic submucosal dissection.
✓ Commercial caps are standardized, rigid, and associated with additional procedural costs.
✓ Three-dimensional printing enables customizable, low-cost endoscopic accessories.

**WHAT IS NEW HERE**

✓ A 3D-printed distal cap is noninferior to a commercial cap for ESD procedure time.
✓ The 3D-printed cap provides superior visibility, scope control, and tissue traction.
✓ Customizable 3D-printed caps represent a safe and cost-effective alternative for ESD.

## ABBREVIATIONS:

µm: micrometers
3D: three-dimensional
AI: artificial intelligence
CAD: computer-aided design
CAP: distal attachment
EMR: endoscopic mucosal resection
ESD: endoscopic submucosal dissection
EVPS: ex vivo pig stomachs
min: minutes
mm: millimeters
SD: standard deviation
USD: United States dollars
